# Case Report: Precision Medicine Target Revealed by *In Vitro* Modeling of Relapsed, Refractory Acute Lymphoblastic Leukemia From a Child With Neurofibromatosis

**DOI:** 10.3389/fonc.2022.851572

**Published:** 2022-04-20

**Authors:** Susan L. Heatley, Elyse C. Page, Laura N. Eadie, Barbara J. McClure, Jacqueline Rehn, David T. Yeung, Michael Osborn, Tamas Revesz, Maria Kirby, Deborah L. White

**Affiliations:** ^1^Cancer Program, Precision Medicine Theme, South Australian Health & Medical Research Institute, Adelaide, SA, Australia; ^2^Faculty of Health & Medical Science, University of Adelaide, Adelaide, SA, Australia; ^3^Australian & New Zealand Children’s Haematology/Oncology Group, Clayton, VIC, Australia; ^4^Faculty of Science, University of Adelaide, Adelaide, SA, Australia; ^5^Dept of Haematology, Royal Adelaide Hospital, Adelaide, SA, Australia; ^6^Australasian Leukaemia & Lymphoma Group, Richmond, VIC, Australia; ^7^Dept of Haematology & Oncology, Women’s & Children’s Hospital, Adelaide, SA, Australia; ^8^Australian Genomics Health Alliance, Parkville, VIC, Australia

**Keywords:** acute lymphoblastic leukemia, relapsed/refractory ALL, neurofibromatosis, Ph-like ALL, iAMP21-ALL

## Abstract

Children with neurofibromatosis have a higher risk of developing juvenile myelomonocytic leukemia and acute myeloid leukemia, but rarely develop B-cell acute lymphoblastic leukemia (B-ALL). Through *in-vitro* modeling, a novel *NF1* p.L2467 frameshift (fs) mutation identified in a relapsed/refractory Ph-like B-ALL patient with neurofibromatosis demonstrated cytokine independence and increased RAS signaling, indicative of leukemic transformation. Furthermore, these cells were sensitive to the MEK inhibitors trametinib and mirdametinib. Bi-allelic *NF1* loss of function may be a contributing factor to relapse and with sensitivity to MEK inhibitors, suggests a novel precision medicine target in the setting of neurofibromatosis patients with B-ALL.

## Introduction

Neurofibromatosis type 1 (NF-1) is an autosomal dominant disorder that affects approximately 1 in 3,000 individuals globally (1). Clinical features are variable although most individuals will develop café-au-lait macules, skin freckling or Lisch nodules with over 3,000 causative germline variants of the neurofibromatosis (*NF1)* gene identified (1). While approximately 50% are familial cases, the remainder occur sporadically and mutations in splice sites are common (2). These mutations lead to haploinsufficiency of *NF1* and of the resultant tumor suppressor protein neurofibromin, an important negative regulator of RAS signaling (3). Children with NF-1 have a higher risk of developing juvenile myelomonocytic leukemia and acute myeloid leukemia, but rarely develop acute lymphoblastic leukemia (ALL) (4). Through *in-vitro* modeling, we have demonstrated a novel *NF1* frameshift mutation, identified in a child with NF-1 and relapsed/refractory B-ALL that led to leukemic transformation and potentially contributed to relapse in this patient. Furthermore, we have shown that B-ALL cells harboring this mutation are sensitive to MEK inhibitors and offer a novel precision medicine target in the context of relapsed/refractory B-ALL for NF-1 patients.

## Case Report

A 9-year-old male first presented in 2015 with persistent migratory subcutaneous swellings, particularly around the eyes, and multiple bony aches with lytic lesions on bone imaging. He had a high white cell count with eosinophilia (WBC 43.4 × 10^9^/L, eosinophils 23.87 × 10^9^/L). A bone marrow (BM) exam showed 10% lymphoblasts (with phenotype CD10/19/34^+^), without disease in the blood or CSF. Fluorescent *in-situ* hybridization (FISH) subsequently identified iAMP21 ALL. Although previously undiagnosed, NF-1 was clinically suspected due to typical skin changes. This was confirmed with the identification of a previously reported pathogenic (ClinVar VCV000578879.5, ACMG) germline NF1 donor splice site mutation, NM_00267.c.1845G >A: p.L615= (5).

Transcriptomic sequencing (TruSeq Stranded mRNA LT kit, Illumina, CA) was performed on CD19^+^ BM lymphoblasts sorted by flow cytometry. This revealed a *P2RY8–CRLF2* gene fusion, with no other clinically relevant variants, while a custom Taqman low density array indicated the patient also had the high-risk B-ALL subtype Ph-like ALL (6), Multiplex ligation-dependent probe amplification (MLPA) (MRC Holland, Amsterdam, the Netherlands; MLPA SALSA probe mixes P202, P335 and P327) confirmed iAMP21 and identified *IKZF1* exon 2-3 and *BTG1* exon 1-2 deletions.

The child was treated with a modified treatment plan based on the high-risk arm of AIEOP-BFM ALL 2009 with cumulative anthracycline capped at 270 mg/m^2^ due to NF-1 related altered cardiac architecture. At presentation an echocardiogram was performed, with a mass detected in the wall of the right ventricle. This was considered benign and likely a neurofibromatosis lesion and not a congenital defect. The lesion remained stable and left ventricular function remained normal throughout therapy. Pegylated asparaginase was discontinued after five doses due to severe pancreatitis. After two years of treatment, remission was achieved; however, the patient relapsed off treatment six months later (2.5 years after diagnosis). He proved refractory to both salvage chemotherapy and blinatumomab and 3 years after diagnosis, further investigations commenced. These were approved by the Royal Adelaide Hospital Human Research Ethics Committee (HREC/15/RAH/54; RAH Protocol: 150212) and conducted in accordance with the Declaration of Helsinki.

## Results and Discussion

**Figure 1:**
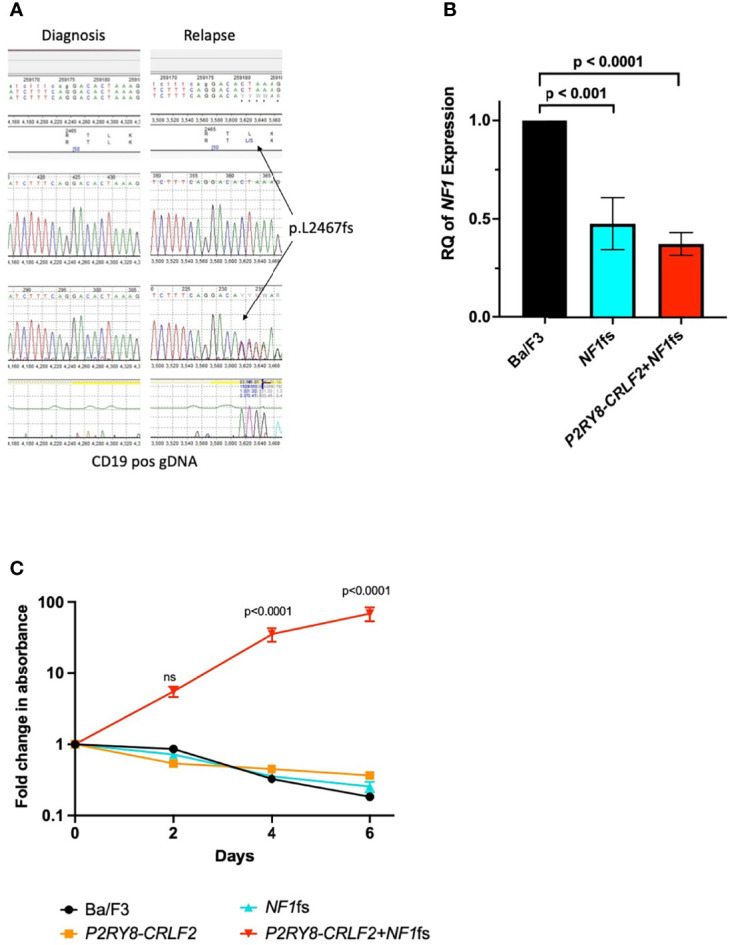
*NF1* p.L2467fs is transformative in Ba/F3 cells with the *P2RY8–CRLF2* gene fusion. **(A)** Sanger sequencing demonstrating the *NF1* p.L2467fs occurs only at relapse in the genomic DNA of the patient. **(B)** qPCR confirming reduced expression of *NF1* in of Ba/F3 cell lines containing the *NF1*fs—blue and *P2RY8–CRLF2* + *NF1*fs—red. RQ, relative quantitation = 2-ddCT, where Ba/F3 (black) was normalized to 1 and each cell line was compared to this control. An unpaired, 2-tailed t-test was used for statistical analysis. **(C)** A proliferation assay demonstrating *P2RY8–CRLF2* + *NF1*fs cells (shown in red) are IL3 independent in comparison to Ba/F3 (black), *P2RY8–CRLF2* (orange) *NF1*fs (blue). Results are from three independent experiments and error bars represent ± SEM, two-way ANOVA (Bonferroni multiple comparisons) was used for statistical analysis. All statistical analyses were performed in GraphPad Prism v9, where p <0.05 was considered significant. Error bars represent ± SEM of three independent experiments. ns, not significant.

The *P2RY8–CRLF2* gene fusion, iAMP21, *IKZF1* exon 2-3 and *BTG1* deletions remained detectable by transcriptomic sequencing and MLPA at relapse without evidence of any additional variants. Whole exome sequencing of CD19^+^ BM cells from diagnosis and relapse as well as mesenchymal stem cells (MSC) (as a germline control) was performed using the SureSelect Clinical Research Exome v2 kit (Agilent Technologies, Santa Clara, CA, USA) and run on the Illumina NextSeq platform. A *NF1* frameshift (fs) mutation at the start of exon 50, NM_000267 c.7398-7399insTC:p.Leu2467SerfsTer2, was identified at relapse, resulting in a premature stop codon and loss of the remaining 8 exons. This was predicted to be damaging by SIFT Indel (7) and was not present at either diagnosis or in MSC and was validated in genomic DNA by Sanger sequencing (**Figure 1A**; **Supplementary Methods**).

To understand the contribution of the *NF1*fs to relapse, the *P2RY8–CRLF2* gene fusion was first transduced into the interleukin 3 (IL3) dependent B-ALL murine cell line Ba/F3 (**Supplementary Methods**). The gene fusion *P2RY8–CRLF2* is thought to be a secondary event in iAMP21 ALL, occurring in approximately 38% of these patients (8). As *P2RY8–CRLF2* is not transforming on its own, modeling with this fusion provides an ideal backbone to determine if the cumulative addition of the *NF1*fs is able to induce leukemic transformation. The corresponding *NF1*fs (equivalent to *Nf1* exon 51 in the mouse and demonstrating over 94% homology to human *NF1*) was then introduced to the Ba/F3 *P2RY8–CRLF2* cells by CRISPR/Cas9 with doxycycline hyclate added to induce the frameshift (**Supplementary Methods**). The presence of the mutation was validated by Sanger sequencing and significantly reduced *NF1* expression was demonstrated by qPCR (**Supplementary Methods**) in both *NF1*fs and *P2RY8–CRLF2* + *NF1*fs lines compared to parental Ba/F3 cells (p <0.001 and p <0.0001, respectively) (**Figure 1B**). A proliferation assay (CellTiter-Glo 2.0^®^, Promega, Madison, WI, USA, **Supplementary Methods**) was performed without the addition of IL3 over six days and demonstrated that the *P2RY8–CRLF2* + *NF1*fs cell line was IL3 independent, indicative of leukemic transformation, whereas all other lines were not (vs Ba/F3, p <0.0001 at days 4 and 6) (**Figure 1C**).

*NF1* p.L2467fs is predicted to undergo non-sense mediated decay (NMD) (SIFT Indel) whereby mRNAs containing premature stop codons and hence altered gene expression are eliminated (9, 10). The inactivation of tumor suppressor genes provides a selective advantage during tumor development allowing proliferation and survival (10). However, a study by Lindeboom et al. found that ~35% of tumor suppressor genes analyzed, including *NF1*, underwent NMD less frequently, likely due to the potential of haploinsufficiency causing cancer (11).

**Figure 2:**
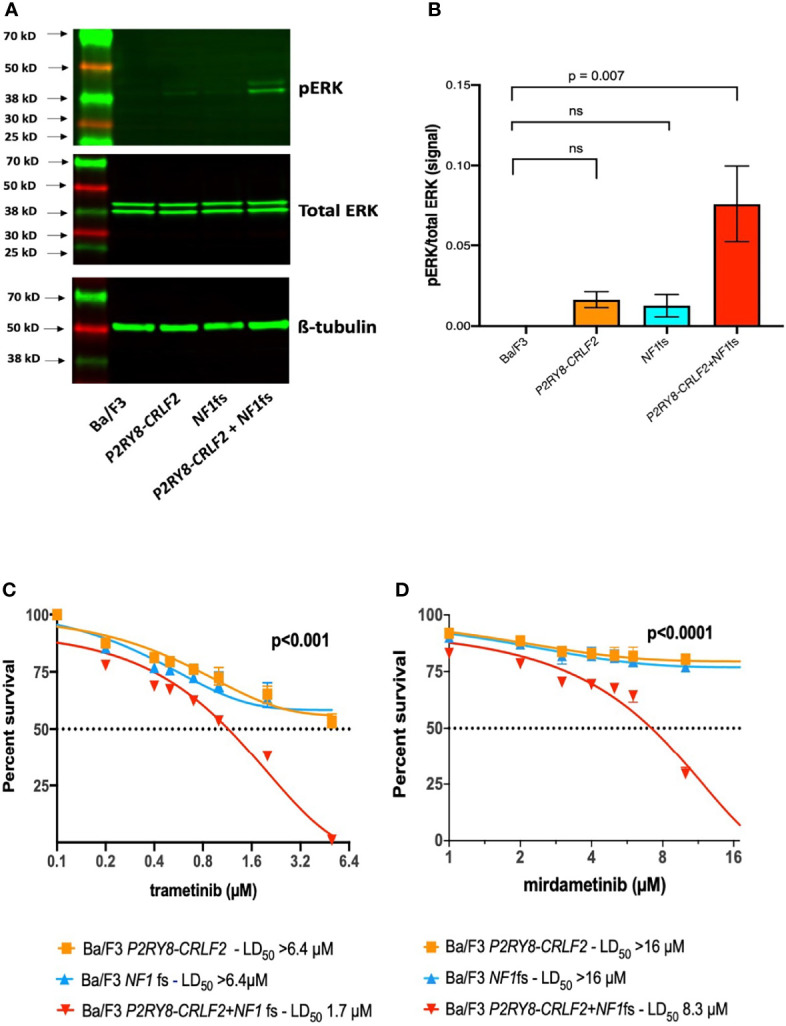
*P2RY8–CRLF2* + *NF1*fs cells demonstrate increased pERK and are sensitive to trametinib and mirdametinib. **(A)** Representative western blotting of pERK, total ERK and ß-tubulin and **(B)** densitometry of three independent experiments of Ba/F3 (black), *P2RY8–CRLF2* (orange), *NF1*fs (blue) and *P2RY8–CRLF2 + NF1*fs (red). Cells were washed ×3 to remove IL3 and allowed to rest for 5 h before lysing. One-way ANOVA (Dunnet’s multiple comparisons) was used for statistical analysis. Sensitivity of Ba/F3 *P2RY8–CRLF2* (orange), *NF1*fs (blue) and *P2RY8–CRLF2 + NF1*fs (red) was assessed following exposure to increasing concentrations of **(C)** trametinib or **(D)** mirdametinib at 3 days. Cell death was measured by flow cytometric analysis of Annexin-V/7-AAD exclusion. Dotted lines across the y-axis denote 50% of the drug concentration required for lethal dose (LD_50_) and x-axis is in log^2^ scale. Two-way ANOVA (Bonferroni multiple comparisons) was used for statistical analysis. All statistical analyses were performed in GraphPad Prism v9, where p <0.05 was considered significant. Error bars represent ± SEM of three independent experiments. ns, not significant.

It has recently been shown that the loss of the nuclear localization signal in exon 51 of *NF1* leads to impaired chromosome congression and accumulation of micronuclei in astrocytes, thereby disrupting the tumour suppressor function of NF1 (12). Furthermore, in cancers, micronuclei are known to provide a clonal advantage in the affected cell (13). Neurofibromin can be constitutively phosphorylated at the C-terminus through an activated c-AMP protein kinase that promotes association with 14-3-3. This association negatively regulates NF1-GAP activity, supressing RAS signaling and inducing cell cycle arrest (14). Taken together, these studies highlight the importance of the C-terminus to loss of function (LOF). Therefore, as NF1 is a negative regulator of RAS and to demonstrate LOF due to the C-terminus *NF1* p.L2467fs, western blotting of pERK, located downstream of RAS, was performed (**Supplementary Methods**). Significant upregulation of pERK was confirmed in the *P2RY8–CRLF2 + NF1*fs cells in comparison to the Ba/F3 control cells (p = 0.007) (**Figures 2A, B**).

NF-1 patients can develop severe morbidities, namely, plexiform neurofibromas and glioma, both as a result of bi-allelic *NF1* loss and increased RAS signaling (1). A number of clinical trials using mTOR or MEK inhibitors are underway to assess efficacy in this setting (1, 15). The second generation MEK inhibitor selumetinib was the first FDA approved drug for use in NF-1 patients with inoperable plexiform neurofibromas, successfully reducing tumor burden, however tumor regrowth was observed when the dosage was reduced or ceased due to side effects (15, 16). A 3-day AnnexinV-7AAD cell death assay with increasing concentrations of selumetinib was performed (**Supplementary Methods**); however sensitivity was not observed in any cell line (data not shown). Furthermore, in a recent study, selumetinib failed to elicit an anti-leukemic response in ALL patient derived xenografts with the *P2RY8–CRLF2* gene fusion (IC_50_ >10 µM) (17).

The third generation MEK inhibitors trametinib (NCT02124772) and mirdametinib (NCT04923126) are in clinical trials for NF-1 patients and, as with selumetinib, have also shown efficacy via *in-vitro* and/or *in-vivo* models of ALL with *RAS* mutations (18–20). As the third generation MEK inhibitors show greater efficacy, a 3-day cell death assay was again performed, and only *P2RY8–CRLF2* + *NF1*fs cells demonstrated sensitivity to trametinib (LD_50_ *P2RY8–CRLF2* >6.4 µM, *NF1*fs >6.4 µM, *P2RY8–CRLF2* + *NF1*fs = 1.7 µM; p <0.001; **Figure 2C**) and mirdametinib (LD_50_ *P2RY8–CRLF2* >16 µM, *NF1*fs >16 µM, *P2RY8–CRLF2* + *NF1*fs = 8.3 µM; p <0.0001; **Figure 2D**). This finding suggests that MEK inhibitors also effectively target increased RAS signalling due to *NF1* mutations in the setting of B-ALL. Selumetinib, as a combination therapy with dexamethasone for ALL or as maintenance therapy in solid tumors, are currently in clinical trials (NCT03705507 and NCT04348045, respectively). Therefore, the potential for trametinib and mirdametinib to also be used as part of these treatment options is appealing to improve outcomes in ALL.

### Conclusion

Here, we have demonstrated a loss-of-function (LOF) *NF1*fs mutation using an *in-vitro* model of ALL. Germline *NF1* haploinsufficiency and a second hit *NF1* mutation in B-ALL is limited to one report of monozygotic twins with neurofibromatosis (21). We propose that *NF1* p.L2467fs together with the germline *NF1* p.L615= caused bi-allelic LOF and therefore contributed to relapse. An understanding of the genomic complexities that lead to relapse may also inform personalized treatment strategies. While the patient subsequently achieved remission with inotuzomab ozogamicin and underwent successful stem cell transplantation, the sensitivity to MEK inhibitors, currently in clinical trials for patients with plexiform neurofibromas (trametinib) or pediatric low-grade glioma (mirdametinib), suggests a novel targeted therapeutic option for neurofibromatosis patients with B-ALL.

## Data Availability Statement

The data presented in the study are deposited in the European Genome Phenome Archive, accession number EGAS00001006187.

## Ethics Statement

The studies involving human participants were reviewed and approved by the Royal Adelaide Hospital Human Research Ethics Committee (HREC/15/RAH/54; RAH Protocol: 150212). Written informed consent to participate in this study was provided by the participants’ legal guardian/next of kin.

## Author Contributions

SLH designed the research. SLH and ECP performed experiments. MO, TR, and MK provided samples and/or acquisition and interpretation of clinical data. SLH, ECP, and DLW analyzed and interpreted data. JR performed biostatistical analysis. SLH wrote the manuscript. LNE, BJMc, and DTY provided critical review of the manuscript. All authors listed have made a substantial, direct, and intellectual contribution to the work and approved it for publication.

## Funding

The authors would like to thank the following for their research support: the National Health and Medical Research Council, Australia (APP1057746, APP1044884); the Channel 7 Children’s Research Fund, Adelaide, SA, Australia; the Leukaemia Foundation, Australia; the Cancer Council of South Australia, Adelaide, SA, Australia; Beat Cancer, Adelaide, SA, Australia. SLH is The Kid’s Cancer Project Postdoctoral Fellow, ECP is the Simone Family Postdoctoral Fellow, LNE is the Peter Nelson Leukaemia Research Fellow.

## Conflict of Interest

DTY received research support from Novartis, Ariad and BMS, Honoraria and Advisory role Novartis. DLW received Research support from Novartis and BMS, and Honoraria from BMS. MO received non-financial support from Amgen.

The remaining authors declare that the research was conducted in the absence of any commercial or financial relationships that could be construed as a potential conflict of interest.

## Publisher’s Note

All claims expressed in this article are solely those of the authors and do not necessarily represent those of their affiliated organizations, or those of the publisher, the editors and the reviewers. Any product that may be evaluated in this article, or claim that may be made by its manufacturer, is not guaranteed or endorsed by the publisher.
