# Supplementary material for: Case Report: Precision Medicine Target Revealed by In Vitro Modeling of Relapsed, Refractory Acute Lymphoblastic Leukemia From a Child With Neurofibromatosis
Source: Front Oncol. 2022 Apr 20;12:851572. doi: 10.3389/fonc.2022.851572 (PMC9065550; doi:10.3389/fonc.2022.851572)
Supplement: Supplementary file 1 [file DataSheet_1.docx]

**Case Report: Precision medicine target revealed by *in-vitro* modelling of relapsed, refractory acute lymphoblastic leukaemia from a child with neurofibromatosis**.

Susan L Heatley^1,2,3^, Elyse C Page^1,4^, Laura N Eadie^1,2^, Barbara J McClure^1,2^, Jacqueline Rehn^1,2^, David T Yeung^1,2,5,7^, Michael Osborn^2,3,6,7^, Tamas Revesz^2,3,6^, Maria Kirby^3,6^ and Deborah L White^1,2,3,4,7,8^

1. Cancer Program, Precision Medicine Theme, South Australian Health & Medical Research Institute, Adelaide, South Australia, Australia
2. Faculty of Health & Medical Science, University of Adelaide, South Australia, Australia
3. Australian & New Zealand Children’s Haematology/Oncology Group, Clayton, Victoria, Australia
4. Faculty of Science, University of Adelaide, South Australia, Australia
5. Dept of Haematology, Royal Adelaide Hospital, Adelaide, South Australia, Australia
6. Dept of Haematology & Oncology, Women’s & Children’s Hospital, Adelaide, South Australia, Australia
7. Australasian Leukaemia & Lymphoma Group, Richmond Victoria, Australia
8. Australian Genomics Health Alliance, Parkville, Victoria, Australia

**Supplementary Methods.**

**Sanger sequencing**

DNA was extracted by phenol-chloroform and Polymerase chain reaction (PCR) was performed with the following primers (NF1-forward-CCAGTGTGGCCCAG GAAAGCTAAGA; NF1-reverse-GGCCGACAGTTGGATAGGTGGCTGC) and Phusion High-Fidelity DNA polymerase (New England Biolabs (NEB), Ipswich, MA, USA), according to the manufacturer’s protocol. The resultant PCR product was confirmed as a single product size via DNA gel electrophoresis, followed by Sanger sequencing. DNA chromatogram results were analysed using the DNA variant analysis software Mutation Surveyor® (SoftGenetics LLC, State College, PA, USA), with the complete CDS reference sequence NM_001042492 utilized to identify the presence of mutations.

**CRISPR/Cas9 *NF1* knockout**

The MSCV-IRES-*P2RY8-CRLF2*-GFP vector was retrovirally transduced into Ba/F3 cells. The Benchling gRNA design tool (Biology Software, 2019, [https://benchling.com](https://benchling.com/)) was used to design a 20 bp gRNA targeting exon 51 of *NF1* with a 5’ *Esp*3I restriction site: 5’- TCCCCACTAAAGGAGACCCAGCCA -3’. The FUCas9Cherry and FgH1tUTG plasmids were a gift from Marco Herold (Addgene, Watertown, MA, USA)^1^. FgH1tUTG vector was digested with *Esp*3I and rSAP (NEB) for 1 hour at 37°C. The complementary gRNAs were phosphorylated at a final concentration of 10 µM using T4 PNK (NEB), then diluted 1:125 with nuclease free water. Five ng/µL of FgH1tUTG vector was digested with 0.8 pmol of diluted gRNA and ligated with T4 ligase overnight (NEB) at 4°C.

Retrovirus or lentivirus was produced by transfecting 1 x 10^6^ HEK293T cells in 5 mL Ba/F3 media containing 5% IL-3 conditioned media in a T25 culture flask with 4 µg of the MSCV-IRES-*P2RY8-CRLF2*-GFP vector, 4 µg of the pEQ-Eco packaging vector and 20 µL lipofectamine (Invitrogen, Carlsbad, CA, USA) or 5.5 µg of the FuCas9mCherry vector and FgH1tUTG gRNA vector, with packaging constructs pMD2.G (2.25 µg), pMDL-PRRE (3.375 µg) and pRSV-REV (1.575 µg) with 30 µL lipofectamine respectively. Viral supernatant was harvested 48 hours post-transfection, clarified by centrifugation and passed through a 0.45 µm filter. Ba/F3 cells at a concentration of 3 x 10^5^/mL were centrifuged at 1800rpm for 1 hour with 30 µg/mL polybrene in 4 mL of viral supernatant in a 6-well plate at room temperature. Cells were washed 24 hours later and sub-cultured in original media. Ba/F3 cells were sorted at a concentration of 1 x 10^7^/mL in 2% FCS/RPMI on a BD FACSAria™ for GFP and mCherry double positive cells then sorted for TSLPR-APC (Invitrogen) at >95% purity. Sorted cells were exposed to aqueous 1 µg/mL doxycycline hyclate (dox) (Sigma-Aldrich, St. Louis, MO, USA) for 72 hours to induce a frameshift mutation.

**qPCR**

RNA was extracted using TRIzol^TM^ reagent (Thermo Fisher Scientific, Waltham, MA, USA) followed by cDNA synthesis incorporating a gDNA elimination step via the QuantiTect reverse transcription kit (Qiagen, Hilden, Germany). Quantitative PCR was performed on the QuantStudio7 (Applied Biosystems) using the following primers (NF1-forward – ATCCCATTCATCACGGTGAC; NF1-reverse – GGTGGCTGCAAGGTATCCTT) and according to the QuantiTect SYBR Green manufacturer’s protocol (Qiagen, Hilden, Germany).

**Proliferation assay**

Ba/F3 cells were seeded at 390 cells/mL in media starved of IL3 in a 24-well plate in duplicate and kept at 37^o^C, in a humidified chamber (5% CO_2_). On days 0, 2, 4 and 6, 20 µL of CellTiter-Glo 2.0® reagent (Promega, Madison, WI, USA) was added to 20 µL of cell suspension. Following 30 min incubation in the dark, luminescence was measured on a Perkin Elmer Victor X5 luminometer set to luminescence at 0.1 seconds.

**Annexin-V-PE/7-AAD exclusion staining protocol**

Following 3 days of drug exposure at increasing concentrations, 48-well tissue culture plates containing 3.5 x 10^4^ cells were centrifuged at 1,400 rpm for 5 min at room temperature (RT). The supernatant was discarded and the cells were resuspended in freshly-prepared ice-cold 1x binding buffer (HBSS + 1% HEPES + 5 mM CaCl_2_), vortexed briefly and samples transferred into a 96-well plate. Cells were then centrifuged at 1,400 rpm for 5 min at RT and the supernatant discarded. Cells were incubated in staining solution (0.4 μL Annexin-V-PE, 0.04 μL 7-AAD, 20 μL binding buffer) for 20 min on ice, in the dark. Following staining, 200 μL of 1x binding buffer was added into each well. Samples were analysed on the BD Canto (BD Biosciences, USA) flow cytometer and data batch-analyzed using FlowJo Software v10 (FlowJo, LLC, USA).

**Western immunoblotting**

Cells were washed x3 to remove IL3 before incubation for 5 hr at 37^o^C, in a humidified chamber (5% CO_2_). Cells were then centrifuged, the supernatant aspirated and pellet resuspended in NP40 lysis buffer (1% Triton X-100, 20 mM Tris-HCl pH 8.2, 150 mM NaCl) in the presence of 10mM ß-glyercol phosphate, 2mM sodium vanadate, 2mM sodium fluoride, 2mM PMSF, 10mM sodium pyrophosphate, 1 µg/mL leupeptin, 5 µg/mL aprotinin and protease (cOmplete^TM^ Mini EDTA-Free Cocktail, Roche, Basel, Switzerland) inhibitors. Protein samples (100 µg) were resolved using 4-15% TGX precast gels (Bio-Rad®, Hercules, CA, USA) and transferred onto PVDF membranes using the Trans-Blot® Turbo™ transfer system (Bio-Rad®, Hercules, CA, USA). Membranes were blocked (Odyssey Blocking Buffer, NE, USA) for 1 hr at RT, and incubated in primary antibody for 72 hr (phospho p44/42 MAPK, anti-rabbit, #4370S, Cell Signaling Technology, MA. USA), or overnight at 4^o^C (total p44/42 MAPK, anti-rabbit, #9102S; ß-tubulin, anti-rabbit, #2128S Cell Signaling Technology, MA., USA) . Immunoblots were washed in 1x TBST for 3 x 5 min prior to incubation in secondary antibodies (donkey-anti-rabbit IRDye-800CW, #926-32213, LiCor Odyssey®, NE, USA) for 1 hour at RT, washed in 1x TBST for 3 x 5 min and visualized on the the LiCor Odyssey®. Data from visualization of Western blots were analysed using the LiCor® ImageStudio^TM^ software.

**Statistical Analyses**

Results were generated using GraphPad Prism v8 (GraphPad Software Inc., USA). Results are from three independent experiments and +/- standard error of the mean (SEM) are shown. LD_50_ values were determined using a sigmoidal dose-response curve, variable slope model on GraphPad Prism. Statistical analysis was performed using a 2-tailed (unequal variance) Student’s t-test or ANOVA where indicated and the differences considered to be statistically significant when the *p* value <0.05.

1. Aubrey BJ, Kelly GL, Kueh AJ, et al. An inducible lentiviral guide RNA platform enables the identification of tumor-essential genes and tumor-promoting mutations in vivo. *Cell Rep*. Mar 2015;10(8):1422-32. doi:10.1016/j.celrep.2015.02.002
